# 

**Published:** 1905-03

**Authors:** 


### CONTENTS.

ORIGINAL COMMUNICATIONS—

THE CAUSES OF APPENDICITIS. By Joseph N. LeConte, M.D., Atlanta, Ga. .... 793

TUBERCULOSIS. By J. R. Briggs, M.D., Dallas, Texas .... 799

THE DOCTOR, THE CHILD AND THE COMMUNITY. By Marion McH. Hull, M.Sc., M.D., Atlanta, Ga. .... 810

THE NEED FOR MEDICAL ORGANIZATION IN GEORGIA. By J. N. McCormack, M.D. .... 820

SOCIETY REPORTS—

PROGRAMME OF THE ATLANTA SOCIETY OF MEDICINE FOR 1905 .... 825

PROGRAMME OF THE AMERICAN ANTI-TUBERCULOSIS LEAGUE FOR THE PREVENTION OF CONSUMPTION .... 832

EDITORIAL NOTES AND COMMENTS—

REORGANIZATION OF THE MEDICAL PROFESSION .... 837

PROPOSED NEW CONSTITUTION AND BY-LAWS .... 839

NEWS AND NOTES .... 846

CONTENTS CONTINUED .... X

### *CONTENTS—CONTINUED.*

SELECTIONS AND ABSTRACTS—

A Study of Eighteen Consecutive Cases of Whooping-Cough Treated by the Elastic Abdominal Belt .... 848
The Early Publication of the Results of Original Research .... 849
Sterility and the X-ray .... 850
The Defectiveness of Water When too Pure .... 851
"Place aux Dames" .... 852
Brewer's Yeast for Pyogenic Infections .... 853
Mission and Function of the Local Medical Journal .... 855
The Sanitary Backsliding of Cuba .... 856
The Significance of a Discharge from the Ear .... 858
Ichthyol in Erysipelas .... 859
Paraffin for Fecal Impaction .... 860
Clinical Observation of 108 Cases of Typhoid Fever .... 861
Hydroleine as a Tissue-Builder .... 861
Municipal Supervision of Restaurants .... 862
Removal of Rust from Steel Instruments .... 862
Inaccurate Clinical Thermometers .... 863
The Nathan Lewis Hatfield Prize for Original Research in Medicine .... 863
Where True Quality is Shown .... 864

MEDICAL ITEMS—CONTINUED .... xvi, xviii, xx, xxii, xxiv, xxvi, xxx

